# A reassessment of Jackson’s checklist and identification of two Down syndrome sub-phenotypes

**DOI:** 10.1038/s41598-022-06984-0

**Published:** 2022-02-24

**Authors:** Chiara Locatelli, Sara Onnivello, Caterina Gori, Giuseppe Ramacieri, Francesca Pulina, Chiara Marcolin, Renzo Vianello, Beatrice Vione, Maria Caracausi, Maria Chiara Pelleri, Lorenza Vitale, Gian Luca Pirazzoli, Guido Cocchi, Luigi Corvaglia, Pierluigi Strippoli, Francesca Antonaros, Allison Piovesan, Silvia Lanfranchi

**Affiliations:** 1 Neonatology Unit, St. Orsola-Malpighi Polyclinic, Via Massarenti 9, 40138 Bologna, BO Italy; 2 Department of Developmental Psychology and Socialisation, University of Padova, Via Venezia 8, 35131 Padua, PD Italy; 3 Unit of Histology, Embryology and Applied Biology, Department of Experimental, Diagnostic and Specialty Medicine (DIMES), University of Bologna, Via Belmeloro 8, 40126 Bologna, BO Italy; 4 Medical Department, Maggiore Hospital, Largo Nigrisoli 2, 40133 Bologna, Italy; 5 Neonatology Unit, St. Orsola-Malpighi Polyclinic, Department of Medical and Surgical Sciences (DIMEC), University of Bologna, Via Massarenti 9, 40138 Bologna, BO Italy; 6 Present Address: Unit of Child Neuropsychiatry, St. Orsola-Malpighi Polyclinic, Department of Medical and Surgical Sciences (DIMEC), University of Bologna, Via Massarenti 9, 40138 Bologna, BO Italy; 7 Present Address: Department of Medical and Surgical Sciences (DIMEC), University of Bologna, Via Massarenti 9, 40138 Bologna, BO Italy

**Keywords:** Genetics, Behavioural genetics, Clinical genetics, Medical genetics

## Abstract

Down syndrome (DS) is characterised by several clinical features including intellectual disability (ID) and craniofacial dysmorphisms. In 1976, Jackson and coll. identified a checklist of signs for clinical diagnosis of DS; the utility of these checklists in improving the accuracy of clinical diagnosis has been recently reaffirmed, but they have rarely been revised. The purpose of this work is to reassess the characteristic phenotypic signs and their frequencies in 233 DS subjects, following Jackson's checklist. 63.77% of the subjects showed more than 12 signs while none showed less than 5, confirming the effectiveness of Jackson's checklist for the clinical diagnosis of DS. An association between three phenotypic signs emerged, allowing us to distinguish two sub-phenotypes: Brachycephaly, short and broad Hands, short Neck (BHN), which is more frequent, and "non-BHN". The strong association of these signs might be interpreted in the context of the growth defects observed in DS children suggesting decreased cell proliferation. Lastly, cognitive assessments were investigated for 114 subjects. The lack of association between the presence of a physical sign or the number of signs present in a subject and cognitive skills disproves the stereotype that physical characteristics are predictive of degree of ID.

## Introduction

Down syndrome (DS) or trisomy 21^1^ is defined by the presence of an extra full or partial copy of chromosome 21^2^. It is the most common human chromosomal disorder and the leading genetic cause of intellectual disability (ID), with an incidence of 1 in ~ 800 live births^3^.

DS phenotype is characterised by several clinical features of which the most constant and typical are ID and craniofacial dysmorphisms, together with other variable signs and symptoms, such as cardiac malformations and growth delay^3–9^. Physicians can suspect DS based on some characteristic physiognomic features of infants and physical examination is the first essential diagnostic evaluation. After visiting 48 newborns with DS, Hall was the first who identified 10 cardinal signs for clinical diagnosis which are easy to assess and occur in over 40% of affected infants. They are unusual in non-DS infants and the same defect is always manifested in the same way. The presence of 6 or more of these signs leads to the presumably correct clinical diagnosis of DS^10^. In 1972, Lee and Jackson drafted a checklist of 25 signs of DS, the so-called Jackson's checklist, observing 150 individuals suspected to be affected^11^. In 1976, Jackson and coll. used these clinical features in a total number of 291 children and identified that individuals with 13 or more signs can correctly be diagnosed as affected by DS, while individuals with less than 5 signs are not-affected^12^. In the other cases, the probability of being affected is then different according to the number of signs found: those with 5 or 6 signs have 23% probability of having DS, 7 to 9 signs 60% probability, 10 to 12 signs 84% probability. In 1980, Fried proposed a simplified score based on 8 signs, including some chosen by Hall and adding others to be used routinely to screen newborns for a suspected DS diagnosis^13^. Despite the enormous spread of prenatal genetic testing and the wide accessibility and rapidity of post-natal testing, similar scores or checklists have rarely been revised, while their utility in improving the accuracy of clinical diagnosis was reaffirmed^14,15^ and supported by several factors. First, in children with a few suspicious signs of DS at birth, being aware of a cut-off to exclude the diagnosis of DS allows a cautious approach, limiting unnecessary anxiety in parents, contrary to what often happens^14,16^. Second, a reasonably confident clinical diagnosis can be provided while awaiting chromosome analysis helping the family to begin to accept the diagnosis at an earlier stage. In addition, the clinical diagnosis is particularly important in the rare cases of partial trisomy, translocations or mosaicism that may not be identified with the normal karyotype and therefore require additional genetic testing. Finally, the diffusion of this checklist is an excellent way to bring attention to the clinical and phenotypic signs of DS contributing to a better understanding of the role of the excess chromosome 21.

Considering current knowledge, ID is the only clinical sign always present in DS individuals, with a specific neurocognitive and neurobehavioral phenotype different compared with that of other syndromes associated with ID. It is characterised by a slower developmental trajectory, which starts to become evident between 6 months and 2 years of age with a delay in the acquisition of the main psychomotor developmental milestones and subnormal neuropsychological testing^17^. Furthermore, ID results associated with typical brain imaging and neural structural correlates, like reduction in brain size and weight, delayed myelination and reduction of neuronal interconnections^18,19^. Typical areas of weakness are verbal processing, expressive language, and problem solving. Some physical features like small mouth and jaws, larger tongues and poor muscle tone hinder the correct pronunciation of sounds. In addition, hearing loss is negatively associated to language development. Despite these difficulties, relative strengths are found in visual processing, receptive language and nonverbal abilities^20,21^. Regarding adaptive functioning, socialisation is the strongest domain, followed by communication and finally daily living skills^22^. According to some studies, intelligence quotient (IQ) values of individuals with DS vary from 35 to 70, indicating mild to moderate ID^23^, while according to others, IQ values vary between 25 and 55, resulting in moderate or severe ID with a mental age rarely exceeding 8 years^24^. Children with DS make steady gains in mental age during childhood and adolescence, but their cognitive development is slower than typically developing children, and therefore their IQ scores tend to drop progressively due to the fact that the gap between mental age and chronological age becomes more pronounced over time^20^.

Being that the DS phenotype is mostly characterised by typical craniofacial dysmorphisms and ID, previous studies attempting to correlate physical characteristics with ID showed contradictory results due to the lack of a systematic approach^25,26^. Thus, the purpose of this work is to reassess the characteristic phenotypic signs and their frequencies in 233 subjects with DS, following the more comprehensive feature list for a DS clinical diagnosis, called the Jackson's checklist^11,12^. Subsequently, we performed a phenotype-phenotype correlation study in order to find statistically significant associations between Jackson’s signs. Finally, the presence of a correlation between clinical features and cognitive assessments carried out in 114 DS subjects was investigated to explore if a physical characteristic might be associated with the degree of ID to better understand the relationship of the global DS phenotype.

## Results

### Phenotypic data

Clinical data were collected from 233 subjects with DS with an overall mean age ± standard deviation (SD) at the time of the visit of 9.78 ± 6.28 years (Supplementary Table 1). Sex distribution was 91 females (F) and 142 males (M).

**Figure 1:**
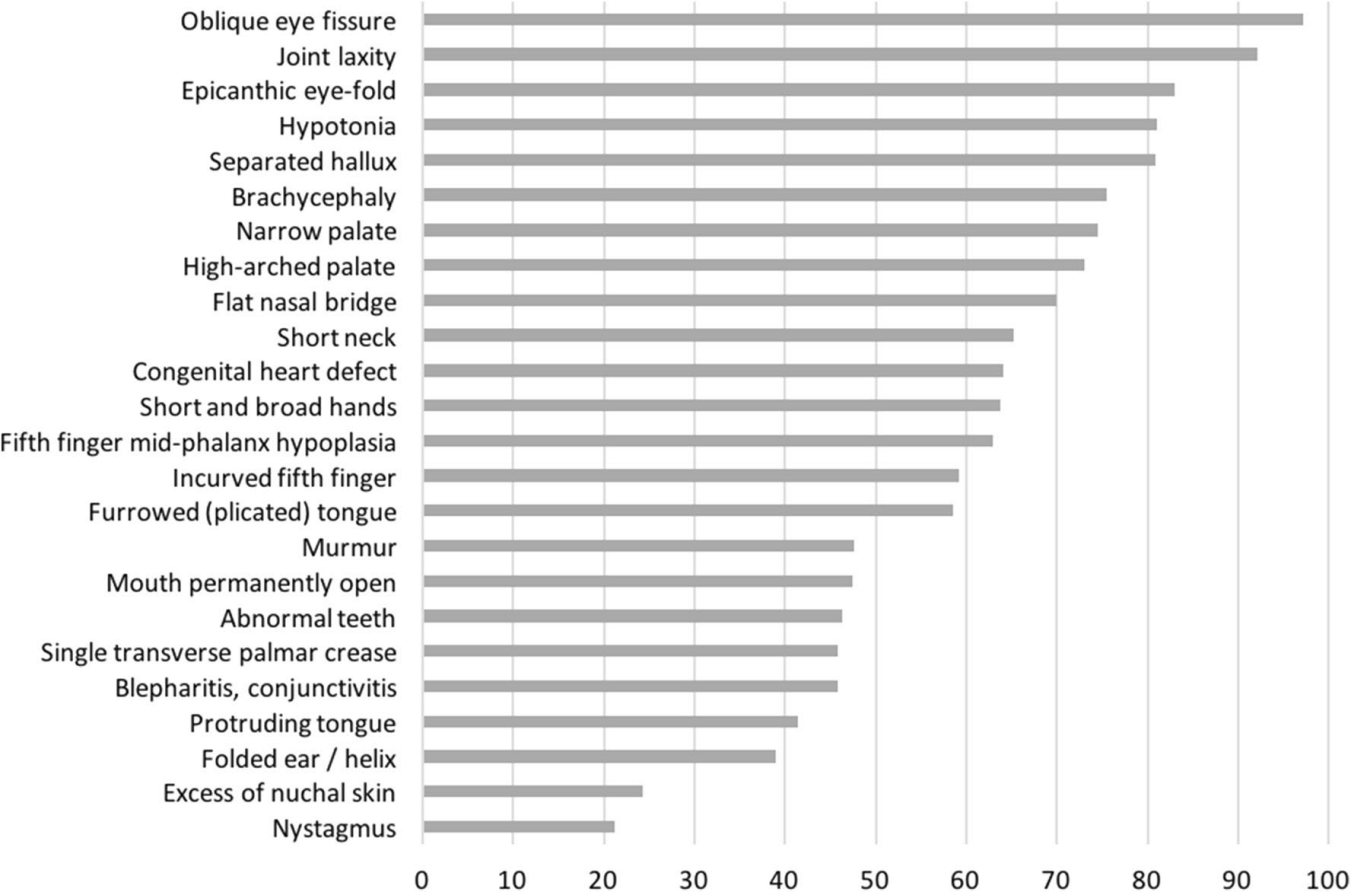
Representation of frequencies of Jackson's signs in our study sample. Brushfield spots was excluded. Details on frequency and number of subjects with available information and with the sign present are in Table 1.

In this study, a reassessment of the characteristic phenotypic signs in all subjects with DS was performed following the more comprehensive feature list proposed for the DS clinical diagnosis^11,12^. Regarding the 25 phenotypic signs of DS described in Jackson's checklist, the brushfield spots feature was not considered (see “Methods” section for details). Considering the remaining 24 features, they are never all present together in a subject, in fact, the maximum is 20 signs present in one child. In addition, there is not a single phenotypic sign always present in all children. The frequency of the 24 phenotypic features in our study samples of 233 children are collected in Table 1 and represented in Fig. 1 as percentage. The comparison of our frequencies with previous studies is shown in Table 2 and represented in Supplementary Fig. 1 as percentage. Both Pearson and Spearman correlation analyses show the best concordance with frequencies presented by Gustavson^27^ and then with frequencies by Jackson et al.^12^, while a weak correlation is found with data by Oster^28^ (Table 3).

**Table 1:** Frequency of Jackson's signs.

Jackson's sign	n	Subjects with sign present	Frequency (%)
Oblique eye fissure	218	212	97.25
Joint laxity	214	197	92.06
Epicanthic eye-fold	205	170	82.93
Hypotonia	174	141	81.03
Separated hallux	210	170	80.95
Brachycephaly	212	160	75.47
Narrow palate	184	95	74.46
High-arched palate	186	136	73.12
Flat nasal bridge	223	156	69.96
Short neck	201	131	65.17
Congenital heart defect	228	146	64.04
Short and broad hands	212	135	63.68
Fifth finger mid-phalanx hypoplasia	205	129	62.93
Incurved fifth finger	213	126	59.15
Furrowed (plicated) tongue	193	113	58.55
Murmur	183	87	47.54
Mouth permanently open	221	105	47.51
Abnormal teeth	177	82	46.33
Single transverse palmar crease	216	99	45.83
Blepharitis, conjunctivitis	199	91	45.73
Protruding tongue	225	93	41.33
Folded ear/helix	208	81	38.94
Excess of nuchal skin	95	23	24.21
Nystagmus	218	46	21.10

Brushfield spots feature was excluded. Signs are sorted by frequency expressed as percentage. *n* number of subjects with available information, *SD* standard deviation.

**Table 2:** Physical sign frequencies in subjects with DS found in the present study compared with previous works.

	Present study (%)	Jackson et al.^12^ (%)	Oster^28^ (%)	Gustavson^27^ (%)
Oblique eye fissure	97.25	85.1	75.0	86.1
Joint laxity	92.06	59.5	47.0	84.8
Epicanthic eye-fold	82.93	78.5	28.0	54.5
Hypotonia	81.03	40.4	21.0	71.7
Separated hallux	80.95	64.4	47.0	87.4
Brachycephaly	75.47	75.2	74.0	80.6
Narrow palate	74.46	67.7	–	75.5
High-arched palate	73.12	67.7	67.0	69.5
Flat nasal bridge	69.96	86.7	59.0	61.6
Short neck	65.17	70.2	39.0	–
Congenital heart defect	64.04	24.7	–	19.0
Short and broad hands	63.68	61.0	69.0	74.7
Fifth finger mid-phalanx hypoplasia	62.93	51.2	57.0	74.0
Incurved fifth finger	59.15	42.9	48.0	52.0
Furrowed (plicated) tongue	58.55	22.3	59.0	43.6
Murmur	47.54	33.0	–	–
Mouth permanently open	47.51	40.4	67.0	59.1
Abnormal teeth	46.33	31.4	71.0	64.8
Single transverse palmar crease	45.83	60.3	43.0	60.2
Blepharitis, conjunctivitis	45.73	22.3	–	45.7
Protruding tongue	41.33	38.0	49.0	38.1
Folded ear/helix	38.94	42.9	49.0	28.0
Excess of nuchal skin	24.21	60.3	–	–
Nystagmus	21.10	17.3	12.0	–

Brushfield spots feature was excluded. Signs are sorted by decreasing frequency found in the present study. Frequencies are expressed as percentages.

**Table 3:** Pearson and Spearman correlation coefficients and *p*-values of physical sign frequencies in subjects with DS found in the present study compared with previous works.

	^12^ (%)	^28^ (%)	^27^ (%)
Pearson coefficient	0.6087	0.2178	0.6628
*p*-value	0.0016	0.0129	0.0014
Spearman coefficient	0.6342	0.0773	0.6977
*p*-value	0.0009	0.7530	0.0006

Frequencies expressed as percentages are taken from Table 2.

To evaluate the effectiveness of the Jackson's checklist for the clinical diagnosis of DS, we considered only those children with genetic diagnosis of DS for whom at least 18 signs were collected (207 subjects): 132 (63.77%) have at least ( ≥) 13 signs and thus a certain clinical diagnosis, 75 (36.23%) have 5 to 12 signs present and none have less than ( <) 5 features present.

### Associations among Jackson’s signs

Contingency tables and applied Fisher's exact test were used to investigate associations between each possible pair of Jackson’s signs (see Table 4, Supplementary Table 2). Considering the large amount of data, we used false discovery rate (FDR) correction on *p*-value and considered associations with *p*-value after FDR correction < 0.05 as significant, and highly significant with *p*-value after FDR correction < 0.01 (Table 4, Supplementary Table 2). For each pair of signs only one of the two cases is reported as representative.

**Table 4:** Significant associations between Jackson’s signs.

Jackson's sign	Jackson's sign	*p*-value	*p*-value after FDR
Narrow palate	High-arched palate	< 0.00001	< 0.00001
Mouth permanently open	Protruding tongue	< 0.00001	< 0.00001
Congenital heart defect	Murmur	< 0.00001	< 0.00001
**Short and broad hands**	**Short neck**	< 0.00001	0.00002
Fifth finger mid-phalanx hypoplasia	Incurved fifth finger	< 0.00001	0.00002
**Brachycephaly**	**Short neck**	< 0.00001	0.00005
**Short and broad hands**	Fifth finger mid-phalanx hypoplasia	< 0.00001	0.00008
**Brachycephaly**	**Short and broad hands**	0.00009	0.00301
Flat nasal bridge	**Short neck**	0.00018	0.00559
Epicanthic eye-fold	**Short neck**	0.00061	0.01682
Protruding tongue	Furrowed (plicated) tongue	0.00114	0.02857
**Brachycephaly**	Flat nasal bridge	0.00132	0.03046
Joint laxity	**Short neck**	0.00154	0.03267

Brushfield spots feature was excluded. Signs are sorted by increasing *p*-value after false discovery rate (FDR) correction. Most recurrent features are highlighted in bold. The whole set of all possible associations is shown in Supplementary Table 2.

Some of the associations are already known in clinical practice, such as narrow palate with high palate (together called ogival palate) and the presence of heart murmur in congenital heart defects. Interestingly, short neck has the highest number of significant associations (with short and broad hands, brachycephaly, flat nasal bridge, epicanthic eye-fold and joint laxity), followed by brachycephaly (with short neck, short and broad hands and flat nasal bridge). Thus, short neck, brachycephaly and broad and short hands are the most recurrent and highly associated features (Table 4). Since these features are frequently present together, different sub-groups were created and subjects were labelled "BHN" (Brachycephaly, broad and short Hands, short Neck) if all three signs were present (86 subjects, Supplementary Table 1), and "non-BHN" if all three were recorded as absent (21 subjects, Supplementary Table 1).

### Cognitive assessments

Cognitive assessment was carried out in the context of the broader project, thus results of cognitive evaluations obtained from 112 subjects have already been analysed to find a possible correlation with metabolite concentrations^29,30^ and with acquisition of fundamental milestones (e.g., acquisition of sitting, walking, babbling and sphincter control)^17^. The subjects are always indicated with the same DS subject code through the literature, while 2 are presented here for the first time.

Cognitive data were collected studying a total of 114 children/adolescents from 3 to 16 years old, 69 males and 45 females. 43 children were from 3 to 6 years and 11 months old and were evaluated with Griffiths-III scales^31^, 71 were from 7 to 16 years old and were evaluated through WPPSI-III (Wechsler Preschool and Primary Scale of Intelligence) scales^32^.

Detailed results about age and IQ values expressed as mean ± SD for the whole study population and for subjects tested with Griffiths-III test and with WPSSI-III scales separately are shown in Table 5.

**Table 5:** Age and intelligence quotient (IQ) values expressed as mean ± standard deviation for the whole study population and for subjects tested with Griffiths-III and with WPSSI-III scales separately.

	n	Total	n	Griffiths-III	n	WPPSI-III
Age (years)	114	8.77 ± 3.95	43	4.58 ± 1.00	71	11.33 ± 2.67
IQ	114	40.30 ± 11.34	43	45.37 ± 10.38	71	37.24 ± 10.84

*n* number of subjects referred to the group column on the right, *IQ* intelligence quotient.

### Correlations between clinical features and cognitive data

Contingency analysis was used to define the statistical significance of the associations between IQ scores and each Jackson’s sign. The sample population was labelled into two subgroups according to whether their IQ score was higher than/equal to or lower than the mean value calculated in all subjects (40.30), obtaining 56 and 58 subjects respectively. None of the Jackson's signs resulted significantly associated with IQ scores, considering *p*-value after FDR correction < 0.05. In addition, for each Jackson's sign the mean IQ score was compared through unpaired t-test between subjects with and without that feature, with no significant *p*-values after FDR correction (Supplementary Table 3). The same analysis was repeated, dividing children evaluated with Griffiths-III and with WPPSI-III with no significant results (Supplementary Table 3).

Considering subjects divided into the two groups BHN (presence of short neck, brachycephaly and broad and short hands, 86 subjects, Supplementary Table 1) and non-BHN (absence of all the three signs, 21 subjects, Supplementary Table 1), contingency analysis with two IQ score subgroups (higher than/equal to or lower than the mean) resulted not statistically significant. Trying to broaden the non-BHN group to include subjects with at least two signs recorded as absent (and the third present or not registered, thus adding 35 subjects for a total of 56, Supplementary Table 1) in order to make it more comparable with the BHN group, the same analysis showed no statistically significant associations considering IQ scores. Mean IQ values were compared through unpaired t-test between BHN and non-BHN groups, with no significant results considering the whole sample or either subject based on cognitive test (Griffiths-III and WPSSI-III separately, Supplementary Table 4). The same analysis was repeated considering the broader non-BHN group, with no significant results except for subjects administered WPPSI-III test who have a mean IQ of 40.70 in the BHN group and 32.58 in the non-BHN group (*p*-value 0.02100, Supplementary Table 4).

**Figure 2:**
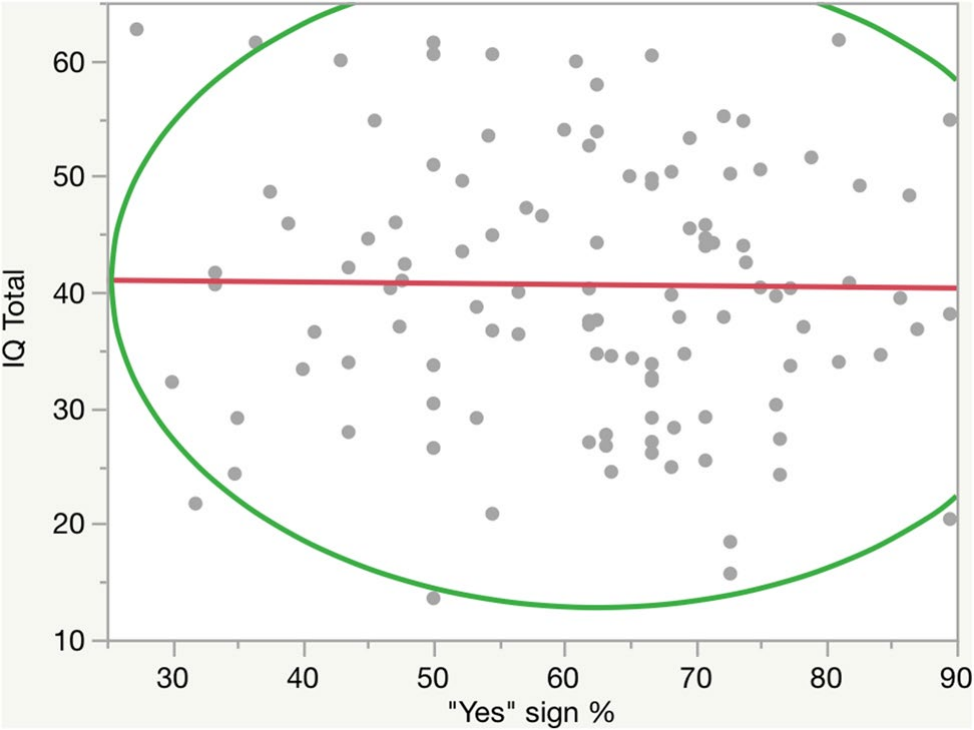
Correlation between the percentage of Jackson’s signs present and IQ scores. Only children for whom cognitive data were available and for whom at least 18 signs were collected (n = 101) were selected.

Considering only those children for whom cognitive data were available and from whom at least 18 signs were collected (101 subjects), the Pearson correlation coefficient between the percentage of Jackson's signs present (calculated on the total of recorded features for each subject) and IQ scores resulted weakly correlated (− 0.01386) with a *p*-value < 0.0001 (Fig. 2). Then, these same 101 subjects were placed into two subgroups according to the presence of at least 13 Jackson's signs and the presence of less than 13 Jackson's signs. Contingency analysis of children with at least or less than 13 Jackson's signs present and IQ scores labelled based on mean IQ threshold did not result statistically significant (*p*-value = 0.6769). Comparing mean IQ values between subjects with at least 13 Jackson's signs or less than 13 through unpaired t-test gave no statistically significant results considering the whole sample or either subject divided based on cognitive test (Griffiths-III and WPSSI-III separately, Supplementary Table 5).

## Discussion

The present study allowed the review of the fundamental typical phenotypic features of DS in 233 children with DS that can be used as an updated reference.

The first objective was to analyse in depth the DS phenotypic characteristics and its presentation patterns following the so-called Jackson's checklist^12^ in order to assess the reliability of the clinical diagnosis and lead to a deeper understanding of the syndrome. Firstly, we have shown that DS signs are never all displayed by the same subject, but present with great variability, and that there is not a phenotypic sign always present in all affected children.

Considering the frequency of the 24 DS characteristic features available in the Jackson's checklist in our sample population, oblique eye fissure and joint laxity are the most frequent signs (frequencies > 90%), followed by epicanthic eye-fold, hypotonia and separated hallux (frequencies > 80%). Comparing our results with those previously published, the best concordance is found with Gustavson^27^ and then with Jackson et al.^12^, while the worst correlation is found with data by Oster^28^. This discrepancy is partially due to the subjectivity and variability of the clinical evaluations, partially to random differences between the cohorts studied and partially to differences in age distribution. 56% of the DS subjects evaluated by Jackson et al.^12^ were less than one year old and only 8.2% were over 10 years old, while Oster^28^ included children and adults, and our study includes mainly children over two years old and to a lesser extent young adults. Furthermore, features such as epicanthic eye-fold, abnormal teeth and furrowed (plicated) tongue become more frequent with increasing age^11,23^. However, there is a high degree of correspondence among frequencies reported and oblique eye fissure is one of the most frequent characteristics in all the considered studies.

To evaluate the effectiveness of this checklist for the clinical diagnosis of DS, the lack of objective measurements for some parameters assessed such as hypotonia and joint laxity and the impossibility to gather all 25 signs in every child prompted us to arbitrarily consider only children (207) for whom at least 18 signs had been collected. Our results show the high sensitivity of this diagnostic method as no affected child presents less than 5 signs, so they would all be considered suspicious for DS. According to Jackson et al.^12^, a certain diagnosis based on clinical signs could have been made in 63.77% of the subjects with DS, as they had at least 13 signs present. This percentage would probably have been higher if we had collected all 25 signs in all children.

Some of the sign associations that emerged from our statistical analysis were absolutely predictable, such as narrow palate with high palate and the presence of congenital heart defect and heart murmur. The associations between mouth permanently open and protruding tongue, and protruding tongue with furrowed tongue could both be explained by macroglossia and the small size of the oral cavity, both typical of DS subjects. The association between fifth finger mid-phalanx hypoplasia and incurved fifth finger finds an explanation during the foetal life of DS subjects when the mid-phalanx of the fifth finger undergoes a reduced and altered ossification process resulting in reduced size and abnormal shape compared to healthy foetuses at the same gestational age^33^. Fifth finger mid-phalanx hypoplasia is also significantly associated with the presence of short and broad hands (*p*-value after FDR correction of 0.00008), a correlation previously highlighted^10^.

An interesting strong association was found among short neck, brachycephaly and short and broad hands. Their frequency ranges from 63.68% for broad and short hands to 75.47% for brachycephaly. In the majority of cases when one sign is present the other two are also present, while in a small group of subjects none of these signs is present. This observation prompted us to question the possibility of distinguishing two sub-phenotypes within the DS population, which could be called "BHN" (Brachycephaly, short and broad Hands and short Neck) and "non-BHN". The strong association of these signs might be considered the result of the decreased cell proliferation demonstrated in subjects with DS since foetal age^34^. The dysplastic aspect of the mid-phalanx of the fifth finger itself, mentioned above, could represent a sign of delayed bone development, since it was similar to the mid-phalanx appearance in healthy foetuses at an early stage of development^10,33^. In addition, the majority of analysed trisomy 21 foetuses^33^ showed a delayed and abnormal ossification of some bone segments, including the long limb bones, the mid-phalanx of the fifth finger and the nasal bone; brachycephaly was always associated with one or two of these signs^33^. Reduced cell proliferation is also the basis of growth failure during the second trimester in trisomy 21 foetuses, of the presence of organs with lower volume, weight and cellularity and finally of a reduced incidence of solid tumors in DS adults^35–38^. In particular, the reduced foetal brain weight compared to healthy controls is well described^39^, together with a smaller foetal liver size which is thought to be the cause of low maternal serum levels of α-feto protein, and the reduced thymic development which contributes to dysregulation of the immune system in DS subjects^35^. Based on these data the non-BHN sub-phenotype might be characterised by a higher level of cell proliferation than the BHN sub-phenotype, although the proliferation rate is in any case lower than that of the rest of the population. Impairment of cell proliferation has been demonstrated in several models, e.g., in DS mouse model TS65Dn^40^, in primary fibroblasts from individuals with DS with a reduced proliferation rate compared with euploid cells^41,42^ and in a DS induced pluripotent stem cell (iPSC)-derived mesodermal and endothelial cellular model in which cell cycle regulation was considered a possible cause for poor progenitor cell proliferation^43^. Several genes have been identified as up- or down-regulated in DS and linked to cell proliferative capacity that can be accessed via several cell cycle checkpoint pathways. Some genes regulate DNA damage repair mechanisms, e.g., *USP16* and *S1P* genes up-regulated in DS iPSC-derived stromal cells^43^, or *DYRK1A* that forms a negative feedback loop with p53^44^. *DYRK1A,* which is located on chromosome 21, is also involved in some possible tumor-controlling mechanisms such as promoting cell cycle exit through DREAM complex assembly^45^, controlling the expression of RE1 silencing transcription factor (REST)^46^, which is a tumor suppressor gene for mammary epithelial cell transformation^47^, colon cancer^47^ and lung cancer^48^. Moreover, REST controls the level of MAD2L1, a protein involved in the mitotic spindle assembly checkpoint. Finally, *DYRK1A*^49,50^ as well other chromosome 21 genes such as *ETS2*^51^ seems to be involved in regulation of apoptotic processes. These basic biological features of DS cells have the potential to protect against cancer progression, so it would be interesting to investigate whether there are differences in molecular pathways related to cell proliferation between BHN and non-BHN phenotypes and to verify if the incidence of solid tumors in adulthood is lower in the BHN phenotypes, although this research will be quite difficult given the extreme rarity of cancer cases in these subjects.

Regarding cognitive profile, the older group of children obtained a mean IQ score which is 8.13 points lower than the mean IQ of the younger group, consistent with the fact that, due to their slower cognitive development, the IQ of children with DS tends to drop progressively with increasing age. Despite this, the wide SD confirms the great inter-individual variability. In fact, for each age group there are subjects with mild ID and others with profound ID (IQ < 25).

The lack of significant associations between IQ level (greater or less than 40.30) and the various Jackson’s signs and between IQ scores and number of Jackson’s sign present highlights the impossibility to identify a physical characteristic predictive of greater ID, as found in previous literature^25^. At the same time, the lack of significant associations between IQ level and the percentage of Jackson’s signs present means that the presence of more physical characteristics in the same subject, typical of the syndrome, is not associated with a greater degree of ID. These analyses might be affected by the incompleteness of recorded features, which we tried to partially avoid by selecting subjects from whom at least 18 signs were collected. These findings are consistent with previous literature results^52^ and with a careful review showing that previous attempts supporting the correlation between physical characteristics and ID were null or contradictory^25^.

Interestingly, only for subjects administered WPPSI-III, the BHN group have a mean IQ of 40.70, while the broader non-BHN group have a mean of 32.58 (*p*-value 0.02100, Supplementary Table 4). These results are also very important from a social point of view, as it has been shown that the more a child's face was perceived as typical of DS, the more the subject was judged to be less intelligent^53,54^.

In conclusion, our review of Jackson's signs in a large number of children with DS provides great statistical significance to the analyses and documents their current validity for clinical use. It will therefore be essential to repeat the evaluations using instruments that allow an objective and repeatable measurement of the data and to complete the collection of all the signs. An in-depth statistical analysis has allowed us to detect typical associations between certain signs, identifying two sub-phenotypes characterised by the presence or absence of the three signs: brachycephaly, broad and short hands and short neck. This discovery can be interpreted in the context of the growth defects observed in DS children. Further studies are therefore necessary to investigate the mechanisms involved in this phenotypic difference, providing a better understanding of the numerous factors involved in determining reduced cell proliferation and important advances in the understanding of DS and cancer research. Furthermore, the presence of a particular sign or the number of physical signs present does not appear to be related to cognitive skills as measured by the tests used. An effort should be made by the general population and in particular professional caregivers to avoid an excessive reduction in educational goals, encouraging the child to achieve cognitive skills in line with his or her potential, regardless of physical characteristics. Finally, it is fundamental to accompany these clinical evaluations with other genetic, epigenetic, molecular and metabolic evaluations to better understand the origin of the phenotypic variability that this syndrome shares with many other syndromes of genetic origin.

## Methods

### Enrollment and ethics approval

The present study was approved by the independent Ethics Committee of the University Hospital St. Orsola-Malpighi Polyclinic, Bologna, Italy (approval no. 39/2013/U/Tess). For all participants involved in the present study, written informed consent was obtained from the subjects themselves if over 18 years of age or parents and/or legal guardians if under 18 years of age, according to the approved protocol for the collection of clinical data. All methods were performed in accordance with the Ethical Principles for Medical Research Involving Human Subjects of the Helsinki Declaration.

Participant enrollment was performed at the Unit of Neonatology of St. Orsola-Malpighi Polyclinic in Bologna, Italy, from February 2014 to February 2020 in the context of the routine follow up provided for DS. A total of 233 children/young adults matching inclusion criteria of diagnosis of DS with homogeneous or mosaic (2 subjects) trisomy 21 and age > 2 years were recruited.

### Clinical data

Children and young adults were evaluated during clinical follow up visits at the Unit of Neonatology of St. Orsola-Malpighi Polyclinic in Bologna, Italy. By filling in a specific form in which the English standard medical terminology was used, clinical data containing anonymous personal, diagnostic, clinical, auxological information from both the neonatal period and the time of the visit were obtained and then reported in an Excel spreadsheet (Supplementary Table 1) in which the cognitive data obtained as explained below were added.

### Evaluation of Jackson's signs

In this study we focus specifically on phenotypic features present in the Jackson's checklist^11,12^, with the exception of brushfield spots (also indicated as iris spots) as it was not possible during routine visits to make the correct evaluation which requires near-infrared light^55^. The feature abnormal teeth was recorded as present if conical teeth and/or small teeth were observed. Features like brachycephaly, plagiocephaly, high-arched palate, narrow palate and oblique eye fissures were only evaluated clinically through the observation of expert physicians. When in doubt, a consensus was reached by at least two physicians.

The collection of all signs was not always possible in all children due to doubtful presence of the feature, due to the lack of medical reports for signs such as nystagmus or hypotonia at birth and because of the young age of the subjects, which leads us to choose a respectful attitude without forcing the children.

Frequency of the phenotypic features is reported, also in comparison with previous studies.

Following Jackson’s 1976 study^12^, in order to evaluate the effectiveness of the Jackson's checklist for the clinical diagnosis of DS, the group of subjects for whom at least 75% (18 over 24) of signs were collected was arbitrarily selected and the number and the percentage of children with at least ( ≥) 13 signs present, with less than ( <) 5 signs present, and the number of children included in the overlap area with a number of signs present between 5 and 12 are reported.

### Cognitive evaluation

From October 2017 to January 2020, a cognitive assessment was also carried out for 114 children from ages 3 to 16 years at the Department of Developmental Psychology and Socialisation, University of Padova, Padova, Italy.

In order to avoid floor effect and have a more sensitive tool of measurement, tests that were more appropriate for the expected mental age than for chronological age of older DS subjects were used^56^. The cognitive profile of 43 DS subjects between 3 and 6 years and 11 months of age was assessed using the Griffiths-III scale^31^. This is an instrument internationally and nationally used for the assessment of the development of the basic functions of the child, providing a general development quotient and specific scores for five subscales, two of which were considered for the purpose of the present study: foundations of learning (A scale) which assesses critical aspects of learning during the early childhood years, and language and communication (B scale), measuring overall language development, including expressive language, receptive language, and, to a lesser extent, use of language to communicate socially with others.

Seventy-one DS subjects from 7 to 16 years old were assessed using the WPPSI-III scale^32^ that consists of different subtests, summarised in three principal indexes: Verbal, Non Verbal and Total. The indexes were composed by the 5 subtests that all participants were able to understand and perform (Block Design, Information, Receptive Vocabulary, Object Construction and Picture Naming). These subtests allowed the computation of the indexes: Verbal (Information + Receptive Vocabulary + Picture Naming), Non Verbal (Block Design + Object Construction) and Total (the mean of Verbal and Non Verbal Index).

For each subtest of both Griffiths-III and WPPSI-III tests, raw scores were registered and later converted to age equivalent (AE) scores. The IQ score was calculated as the ratio of the child’s AE to his/her chronological age, multiplied by 100.

The results of cognitive evaluations obtained from 112 subjects have already been shown in previous papers of Antonaros^29,30^ and Locatelli^17^. These data are indicated with the same DS subject code throughout the literature, while 2 are presented here for the first time.

### Statistical analyses

We performed all statistical analyses with JMP software (SAS Institute, version 14). When necessary, FDR (False Discovery Rate) correction using the Benjamini–Hochberg Procedure^57^ was then performed on all the obtained *p*-values with the FDR Add-in available for JMP software (https://community.jmp.com/t5/JMP-Add-Ins/False-Discovery-Rate-PValue/ta-p/21353). We considered associations with *p*-value after FDR correction < 0.05 as significant, and highly significant with *p*-value after FDR correction < 0.01.

The frequencies expressed as percentage of the phenotypic features calculated in our study samples were compared with frequencies found in previous studies through bivariate statistical analyses.

In order to identify associations between each possible pair of Jackson’s signs, we used 2 × 2 contingency tables with Fisher's exact test and we calculated a two-tailed *p*-value. The whole set of association *p*-values was checked by FDR correction.

For the study of IQ scores and their association with each Jackson’s sign, we divided the children into two subgroups according to whether their IQ score was higher than/equal to (H group) or lower than (L group) the mean value calculated in all subjects. We used contingency analysis and Fisher's exact test to define the statistical significance of the associations. All associations were checked by FDR correction.

Unpaired t-test was used to test if IQ means were significantly different between subjects with and without each Jackson’s feature considering the whole sample or either subject based on cognitive test (Griffiths-III and WPSSI-III separately).

Finally, for the correlation between the number of Jackson’s signs that are present and IQ scores, we considered only subjects for whom at least 18 signs were collected. The percentage of signs present for each child was calculated in order to better reflect the number of present signs out of the total number of signs collected for each child. For this analysis, we used bivariate analysis calculating the Pearson correlation coefficient. Then, in the same subgroup of subjects for whom at least 18 signs were collected, the label "Yes" was used if the number of Jackson's signs present is at least 13 (and "No" otherwise) and the association of this characteristic and IQ score, divided as described above, was tested with contingency analysis and Fisher’s exact test. Unpaired t-test was used to test if IQ means were significantly different between subjects with at least 13 Jackson's signs or less than 13 considering the whole sample or either subject based on cognitive test (Griffiths-III and WPSSI-III separately).

## Supplementary Information

Supplementary Figure 1.

Supplementary Legends.

Supplementary Table 1.

Supplementary Table 2.

Supplementary Table 3.

Supplementary Table 4.

Supplementary Table 5.

## Data Availability

The dataset generated and analysed during the current study has been made available as Supplementary Table 1.
