# Supplementary figures and images for: A reassessment of Jackson’s checklist and identification of two Down syndrome sub-phenotypes
Source: Sci Rep. 2022 Feb 24;12:3104. doi: 10.1038/s41598-022-06984-0 (PMC8873406; doi:10.1038/s41598-022-06984-0)

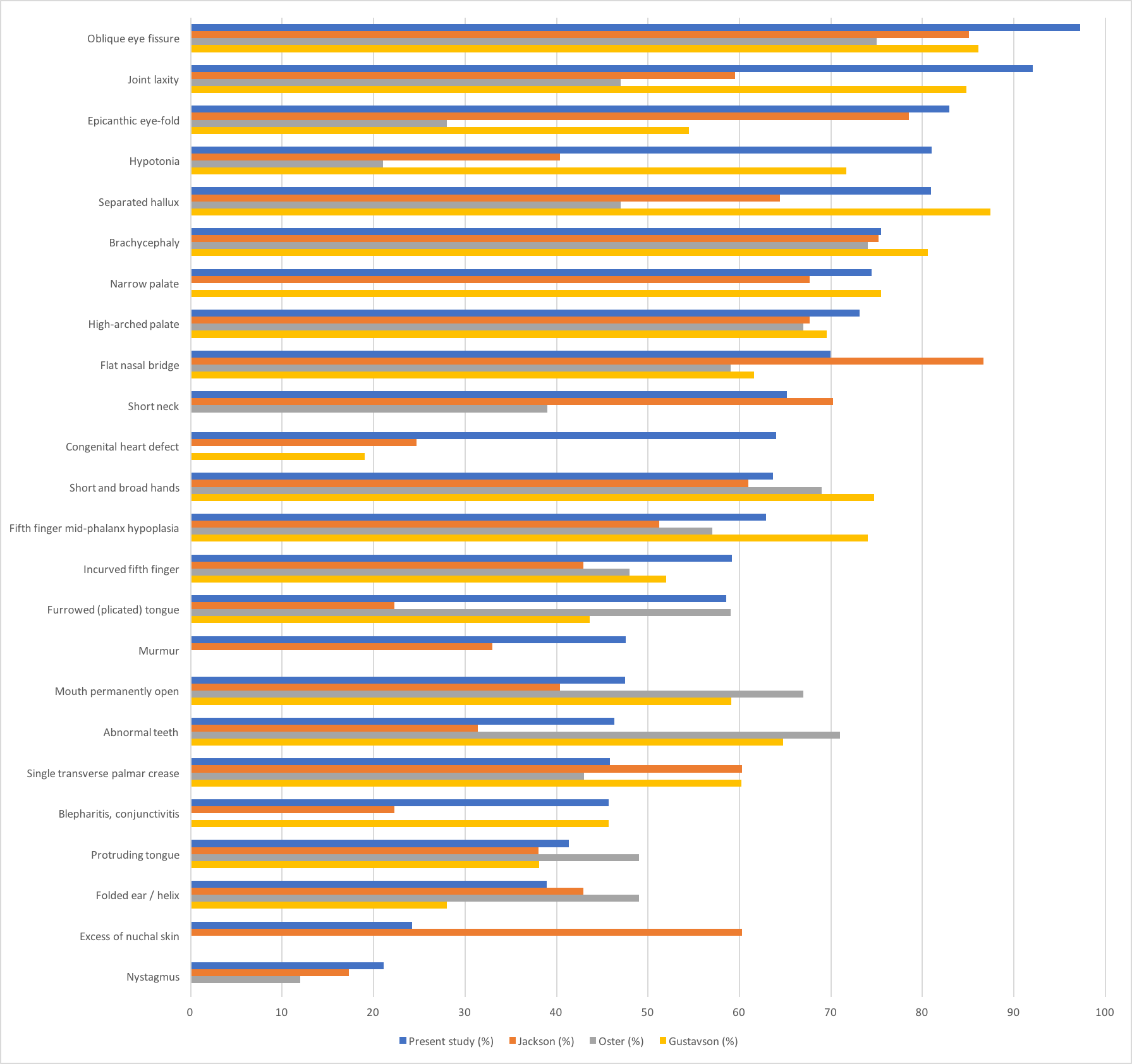

Supplement: Supplementary file 1 — Supplementary Figure 1. [file 41598_2022_6984_MOESM1_ESM.png]
