# Supplementary material for: A reassessment of Jackson’s checklist and identification of two Down syndrome sub-phenotypes
Source: Sci Rep. 2022 Feb 24;12:3104. doi: 10.1038/s41598-022-06984-0 (PMC8873406; doi:10.1038/s41598-022-06984-0)
Supplement: Supplementary file 2 — Supplementary Legends. [file 41598_2022_6984_MOESM2_ESM.docx]

**Supplementary Information**

**Supplementary Figure 1. Comparison of frequencies of Jackson's signs in our study sample with previous studies**. Brushfield spots was excluded. Details on frequency and number of subjects with available information and with the signs present are in Table 2.

**Supplementary Table 1. Clinical data collected for 233 subjects with Down syndrome (DS). "Griffiths-III"**: cognitive test administrated to subjects with DS from 3 to 6 years and 11 months; **"WPPSI-III**": cognitive test administrated to subjects with DS from 7 to 16 years; **"IQ"** Intelligence Quotient; **"Sub-phenotype labeling"**: considering the three signs Brachycephaly, broad and short Hands, short Neck, subjects were labelled "BHN" if all three signs are present and "non-BHN" if all three are recorded as absent. **"Broader sub-phenotype labeling"**: considering the three signs Brachycephaly, broad and short Hands, short Neck, subjects were labelled "BHN" if all three signs are present and "non-BHN" if at least two signs are recorded as absent (and the third present or not registered); **"N/A"**= data not available. As for the subjects highlighted in orange, information was available for less than 18 signs (**"Not empty"** < 18).

**Supplementary Table 2. Correlations between each possible couple of Jackson’s signs.** Brushfield spots was excluded. Contingency tables with Fisher's exact test and calculation of a two-tailed p-value were used. False discovery rate (FDR) correction (Benjamini-Hochberg) was applied to p-value results.

**Supplementary Table 3. Analysis of mean intelligent quotient (IQ) scores for each Jackson's sign.** Unpaired t-test was used to compare mean intelligent quotient (IQ) score for each Jackson's sign between subjects with and without that feature. Brushfield spots was excluded. False discovery rate (FDR) correction (Benjamini-Hochberg) was applied to p-value results. The analysis was performed on whole subject sample and on subjects based on administered cognitive test (Griffiths-III and WPSSI-III). Significant p-value (≤0.05) is highlighted in red.

**Supplementary Table 4. Analysis of mean intelligent quotient (IQ) scores in BHN and non-BHN groups.** Unpaired t-test was used to compare mean intelligent quotient (IQ) score in subjects selected depending on the presence of all the three features, short neck, brachycephaly and broad and short hands (BHN group, 86 subjects) and the recorded absence of all three signs (non-BHN group, 21 subjects). The analysis was performed on whole subject sample and on subjects based on administered cognitive test (Griffiths-III and WPSSI-III). The same analysis was repeated considering the broader non-BHN group (subjects with at least two signs recorded as absent and the third present or not registered, thus adding 35 subjects for a total of 56). Significant p-value (≤0.05) is highlighted in red.

**Supplementary Table 5. Analysis of mean intelligent quotient (IQ) scores in subjects with at least 18 Jackson's signs.** Unpaired t-test was used to compare mean intelligent quotient (IQ) score selecting subjects with at least 18 Jackson's signs recorded based on presence of at least 13 signs or less than 13 signs. The analysis was performed on whole subject sample and on subjects based on administered cognitive test (Griffiths-III and WPSSI-III).
